# Retraction: Advancing Perioperative Neurocognitive Health: A Critical Review of Predictive Tools, Diagnostic Methods, and Interventional Strategies

**DOI:** 10.7759/cureus.r166

**Published:** 2025-01-28

**Authors:** Swetha Lakshminarayanan, Mohazin Aboobacker, Anureet Brar, Mathew Parackal Manoj, Mostafa Mohamed Elsaid Ismail Elnimer, Aamuktha Marepalli, Krutarth Jay Shukla, Muhammad Sheraz Yousaf, Ahsen Taqveem, Muhammad Junaid Hassan

**Affiliations:** 1 General Surgery, Sri Ramachandra Institute of Higher Education and Research, Chennai, IND; 2 Neurosurgery, Jubilee Mission Medical College and Research Institute, Thrissur, IND; 3 Neurology, Sri Guru Ramdas Institute of Medical Sciences and Research, Amritsar, IND; 4 General Surgery, Government Medical College Kottayam, Kottayam, IND; 5 Gastroenterology, Kafr Elsheikh Liver Research Center, Kafr El-Sheikh, EGY; 6 Medicine, Sri Venkateswara Institute of Medical Sciences, Tirupati, IND; 7 Medicine, Gujarat Cancer Society Medical College Hospital and Research Centre, Ahmedabad, IND; 8 Medicine, Faisalabad Medical University, Faisalabad, PAK; 9 Microbiology, Government College University Faisalabad, Faisalabad, PAK; 10 Internal Medicine, Faisalabad Medical University, Faisalabad, PAK

This article has been retracted by the Editors-in-Chief. Numerous inconsistencies have been found throughout the text, including citations that do not match the content. As a result of these inconsistencies, the veracity of this work is now in question and the decision has been made to retract as a means of preserving the scientific record.

